# GFAP as a Potential Biomarker for Alzheimer’s Disease: A Systematic Review and Meta-Analysis

**DOI:** 10.3390/cells12091309

**Published:** 2023-05-04

**Authors:** Ka Young Kim, Ki Young Shin, Keun-A Chang

**Affiliations:** 1 Department of Nursing, College of Nursing, Gachon University, Incheon 21936, Republic of Korea; 2 Neuroscience Research Institute, Gachon University, Incheon 21565, Republic of Korea; 3 Bio-MAX Institute, Seoul National University, Seoul 08826, Republic of Korea; 4 Department of Pharmacology, College of Medicine, Gachon University, Incheon 21936, Republic of Korea; 5 Bio-Medical Sciences, Gachon Advanced Institute for Health Sciences and Technology, Gachon University, Incheon 21936, Republic of Korea

**Keywords:** Alzheimer’s disease, cognitive impairment, glial fibrillary acidic protein, GFAP, blood biomarker

## Abstract

Blood biomarkers have been considered tools for the diagnosis, prognosis, and monitoring of Alzheimer’s disease (AD). Although amyloid-β peptide (Aβ) and tau are primarily blood biomarkers, recent studies have identified other reliable candidates that can serve as measurable indicators of pathological conditions. One such candidate is the glial fibrillary acidic protein (GFAP), an astrocytic cytoskeletal protein that can be detected in blood samples. Increasing evidence suggests that blood GFAP levels can be used to detect early-stage AD. In this systematic review and meta-analysis, we aimed to evaluate GFAP in peripheral blood as a biomarker for AD and provide an overview of the evidence regarding its utility. Our analysis revealed that the GFAP level in the blood was higher in the Aβ-positive group than in the negative groups, and in individuals with AD or mild cognitive impairment (MCI) compared to the healthy controls. Therefore, we believe that the clinical use of blood GFAP measurements has the potential to accelerate the diagnosis and improve the prognosis of AD.

## 1. Introduction

Alzheimer’s disease (AD) is a progressive neurodegenerative disorder, clinically manifested by cognitive deterioration and behavioral disruptions [1]. Over the past few decades, several attempts have been made to identify accurate blood biomarkers for AD. However, differentiating AD from numerous dementia-causing neuropathologies remains difficult [2,3,4,5]. An optimal blood biomarker for AD would exhibit characteristics, such as reliability, reproducibility, non-invasiveness, ease of measurement, and cost-effectiveness [1]. Moreover, it is very important to discover potential targets for developing molecular markers and verifying their effectiveness in the early detection of AD, ultimately leading to improved treatment approaches and better results for patients [6]. AD, predicted to affect more than 152 million people by 2050, is now recognized as common senile dementia with main pathological hallmarks, such as extracellular neuritic plaques containing amyloid-β peptide (Aβ) and intracellular neurofibrillary tangles composed of hyper phosphorylated tau [7].

Some proteins related to the two biomarkers detected in the peripheral blood are attractive candidates for AD diagnosis [8,9,10,11,12] because they are contained in the amyloid cascade hypothesis, which introduces the idea that Aβ misfolding and deposition are the primary precipitants of AD [4,5]. These proteins include 1) single-type proteins such as Aβ40, Aβ42, Aβ oligomers (AβO), total tau (t-tau), tau phosphorylated at threonine 181 (p-tau 181), tau phosphorylated at threonine 217 (p-tau 217), or tau phosphorylated at threonine 231 (p-tau 231) and 2) combined types, such as the Aβ42/Aβ40 ratio, the ratio of amyloid precursor protein669-771 (APP669-771) to Aβ42 (Aβ42/APP669-771 ratio), the Aβ42/Aβ43 ratio, the t-tau/Aβ42 ratio, or the p-tau 181/Aβ42 ratio [8,9,10,11,12]. However, controversy exists regarding the plausibility of the amyloid cascade hypothesis [3,13,14]. Therefore, many researchers have suggested alternative hypotheses that place other proteins or processes, such as neuroinflammation, as the central initiating mechanisms of AD pathogenesis [5,15,16,17,18].

Apart from the Aβ and tau proteins, recent studies have identified other candidates for AD, such as the neurofilament light (NFL) protein, brain-derived neurotrophic factor (BDNF), clusterin, glial fibrillary acidic protein (GFAP), and neurogranin [9,12,19]. GFAP is an astrocytic cytoskeleton intermediate filament protein. The expression and concentrations of GFAP are higher in areas surrounding Aβ plaques and increased with tau accumulation in the brains of patients with AD [20,21]. In pathological situations, astrocytes undergo a series of morphological and functional alterations collectively referred to as reactive astrocytes and overexpress proteins, such as GFAP [22]. Reactive astrocytes contribute to neuroinflammatory changes in AD by releasing cytokines, inflammatory mediators, nitric oxide, and reactive oxygen species and promoting redox status imbalance [23]. Previous studies have shown that reactive astrocytes can precede early pathological hallmarks of AD, such as Aβ and tau, during disease progression [24,25,26,27,28,29,30].

GFAP has recently attracted attention because of preliminary evidence indicating a better performance of the plasma biomarker than its CSF counterpart in detecting AD pathology [31]. Plasma GFAP more accurately discriminated Aβ-positive from Aβ-negative individuals than CSF GFAP (area under the curve for plasma GFAP, 0.69–0.86; area under the curve for CSF GFAP, 0.59–0.76) [32]. Serum GFAP levels were increased in AD and correlated with the Mini-Mental State Examination (MMSE) score [33]. Therefore, GFAP may be a sensitive blood biomarker for detecting and tracking reactive astrogliosis and Aβ pathology [32]. For this reason, we focused on the relationship between blood GFAP and AD and aimed to evaluate GFAP in the peripheral blood as a biomarker for AD by conducting a systematic review and meta-analysis and discussing the possibility of GFAP as an AD blood biomarker.

## 2. Materials and Methods

### 2.1. Literature Search and Selection Criteria

A literature search was performed on PubMed, Embase, Web of Science, and the Cochrane Library on 19 September 2022. All previous papers written in English were included in this study. This study was conducted in accordance with the Preferred Reporting Items for Systematic Reviews and Meta-Analyses (PRISMA) guidelines [34]. For PubMed, we used the following search terms: (AD [MeSH] or Alzheimer* or “mild cognitive impairment”) and (glial fibrillary acidic protein [MeSH] or GFAP) and (blood, plasma, or serum). The following terms were used for the other databases: Alzheimer* or “mild cognitive impairment” and “glial fibrillary acidic protein” or GFAP, and blood, plasma, or serum. The inclusion criterion was a clinical study using human blood samples to assess the GFAP levels between AD and normal controls, or Aβ-positive and Aβ-negative groups. The human blood GFAP level was measured with single-molecule arrays in most studies. In our study, both the AD and normal control groups, as well as the Aβ-positive and Aβ-negative groups, were classified based on each original study. The Aβ status was determined through Aβ PET imaging. The exclusion criteria were (1) studies with disease groups other than AD; (2) studies that did not include the GFAP level in the analysis group; (3) studies that used cell, tissue, or animal models; and (4) commentaries, letters, editorials, conference abstracts, or reviews.

### 2.2. Data Extraction and Analysis

Two authors (K.Y. Kim and K.-A. Chang) independently reviewed the articles, and disagreements were resolved via a discussion by all authors. Among the 1797 studies, 31 were selected based on the title, abstract, and full text. The following data were extracted from the selected studies: first author’s last name, year, country of participants, analyzed group, total sample size by dividing female and male, mean age, blood sample type, and GFAP level in the analyzed group. For the meta-analysis, the standardized mean difference (SMD) of the GFAP level was analyzed between the AD and normal control groups or the Aβ-positive and Aβ-negative groups using comprehensive meta-analysis software version 3 (Biostats Inc., Englewood, NJ, USA). After analyzing the Q statistic and *I*^2^ method to assess the heterogeneity, a random-effects model was used. Publication bias was evaluated using funnel plots and Egger’s intercept test. Statistical significance was defined as *p* < 0.05.

## 3. Results

### 3.1. Characteristics of the Selected Studies

**Figure 1:**
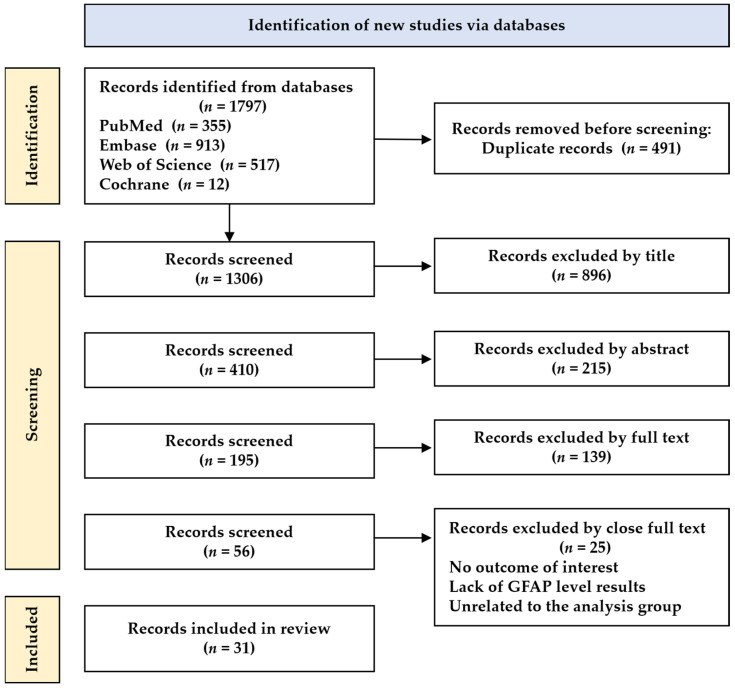
Flowchart of the literature search.

Figure 1 illustrates the literature search and selection process. A total of 1797 studies were screened, including 355 from PubMed, 913 from Embase, 517 from Web of Science, and 12 from the Cochrane databases. After removing duplicates and irrelevant studies, 31 articles were finally subjected to data extraction. Table 1 presents the articles evaluating the blood GFAP levels in patients with AD. In total, 87.5% of the selected articles were published in the last two years, 2021–2022. The participants were from various countries, including the USA, Canada, Spain, France, Italy, Germany, Australia, the UK, Sweden, the Netherlands, Finland, Greece, and Poland. The groups used in the analysis were controls (healthy controls, cognitively unimpaired individuals, etc.), and AD, and Aβ-negative and Aβ-positive groups. The sample sizes of the case and control groups ranged from 8 to 1439. The patients’ sexes and mean ages for each included study are illustrated in Table 1. The GFAP plasma or serum levels were presented in pg/mL in the control and case groups.

### 3.2. Association between GFAP and AD or Aβ-Positive Group

**Figure 2:**
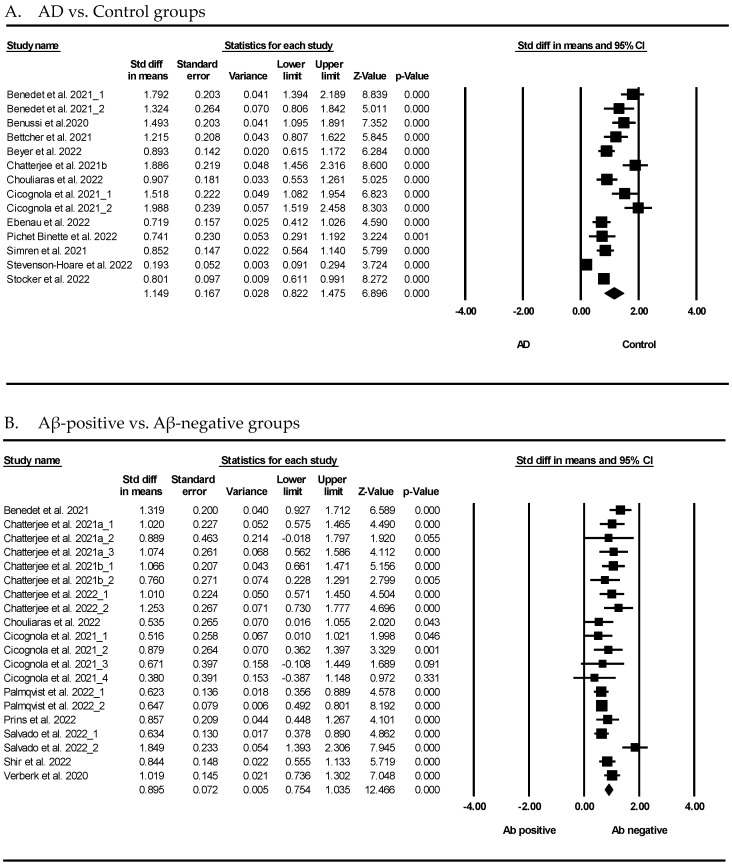
Forest plots of the GFAP levels in the blood of subjects with Alzheimer’s disease (AD) and those who tested positive for Aβ. (**A**) GFAP levels in AD and control, (**B**) GFAP levels in Aβ-positive and Aβ-negative subjects. The forest plots describe the statistical parameters and effect size of each comparison, and quantitatively synthesized results. Black square represents the odds ratio, as calculated for each study and horizontal bars show the 95% CI of each study. The black rhombus represents the confidence limits. GFAP: pg/mL, Std diff: standard difference, CI: confidence interval.

Figure 2 presents the results of the meta-analysis, demonstrating the association between GFAP and AD. As shown in Figure 2A, the GFAP levels showed that patients with AD had a significant difference compared to the normal controls (SMD = 1.149, 95% confidence interval (CI) = 0.822 to 1.475, *p* < 0.001). The random-effects model was used because the heterogeneity was significant (*I*^2^ = 94.32%, *p* < 0.001). Furthermore, GFAP showed that the Aβ-positive group had a significant difference compared to the Aβ-negative group in Figure 2B (SMD = 0.895, 95% CI = 0.754 to 1.035, *p* < 0.001). Considering the heterogeneity, a random-effects model was used (*I*^2^ = 59.76%, *p* < 0.001).

### 3.3. Association between GFAP and Mild Cognitive Impairment (MCI)

**Figure 3:**
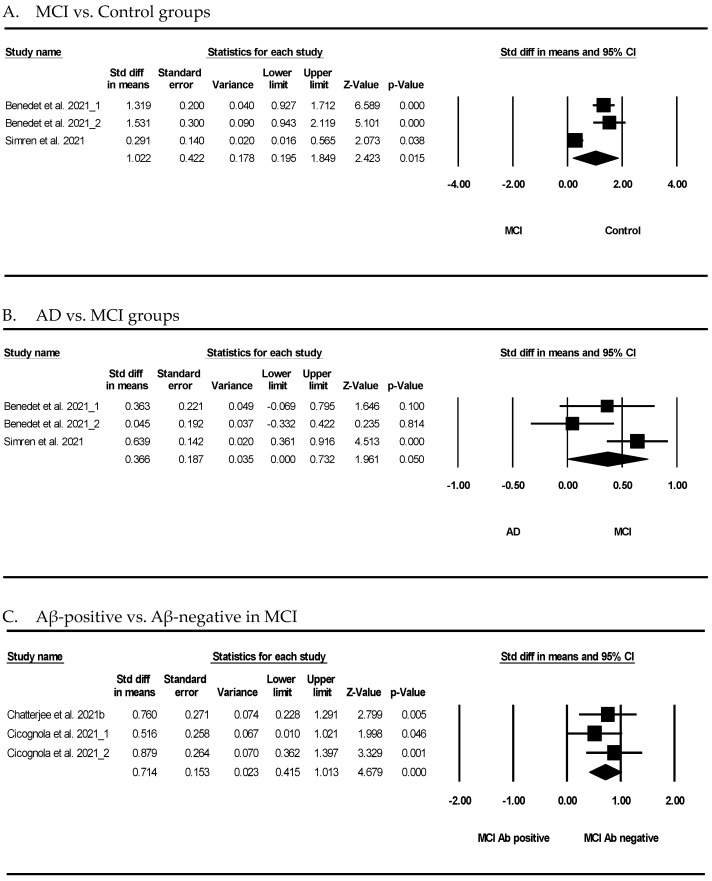
Forest plots of the GFAP levels in the blood of subjects with mild cognitive impairment (MCI). (**A**) GFAP levels in MCI and control, (**B**) GFAP levels in AD and MCI, and (**C**) GFAP levels in Aβ-positive and Aβ-negative subjects. The forest plots describe the statistical parameters and effect size of each comparison, and quantitatively synthesized results. Black square represents the odds ratio, as calculated for each study and horizontal bars show the 95% CI of each study. The black rhombus represents the confidence limits. Std diff: standard difference, CI: confidence interval.

Figure 3 shows the results of the meta-analysis for the potential association between GFAP and MCI. As shown in Figure 3A, the meta-analysis of GFAP showed that the participants with MCI had a significant difference compared to the normal controls (SMD = 1.022, 95% CI = 0.195 to 1.849, *p* = 0.015). GFAP showed that AD was not significantly different compared to MCI (SMD = 0.366, 95% CI = 0.000 to 0.732, *p* = 0.050) (Figure 3B). Furthermore, GFAP showed that the Aβ-positive subjects had a significant difference compared to the Aβ-negative subjects in MCI (SMD = 0.714, 95% CI = 0.415 to 1.013, *p* < 0.001) (Figure 3C).

## 4. Discussion

Blood biomarkers can be used as diagnostic, prognostic, and disease-monitoring tools for AD [14,64,65]. The utilization of biomarkers from peripheral blood presents several benefits for AD diagnosis, including minimal invasiveness, ease of sampling, cost-effectiveness, time-efficiency, and widespread adoption [14,64,65]. Although developing blood diagnostic assays for AD remains challenging owing to the lack of specific biomarkers, methodological inconsistencies, and insufficient standardization of assays, it is an interesting field where many theoretical and clinical screening approaches can be realized [66,67,68,69].

GFAP is a blood biomarker that has recently gained attention in AD research. GFAP is primarily found in astrocytes, a type of glial cell in the brain. GFAP is expressed at a low level in healthy people but is markedly elevated in the brains of patients with AD. Several studies have investigated the potential of GFAP as a biomarker for AD, with promising results. A systematic review and meta-analysis demonstrated that GFAP might be a potential blood biomarker for AD. As shown in Figure 2, the blood GFAP levels in the AD group were significantly higher than those in the control group. GFAP was not only detectable in the blood of AD patients but also discriminated between the AD and control groups. There are several advantages to GFAP as a useful biomarker for AD. First, the use of blood GFAP as an AD biomarker has increased because the possibility of more sensitive assays has made it available for measurement in the blood. As detection techniques, such as the state-of-the-art single-molecule array (Simoa), have advanced quickly, accumulated results have verified that GFAP can diagnose AD patients. Interestingly, many studies have reported that plasma GFAP distinguished between patients with AD and cognitively normal individuals during the last three years (Table 1). It has been revealed that plasma GFAP concentrations correlate strongly with cerebral Aβ pathology [5,40,63]. The rate of change in GFAP concentrations was significantly higher among individuals who developed clinical AD than those with no cognitive impairment [70]. In our meta-analysis, the GFAP levels in patients with AD were also significantly higher than those in normal individuals. This evidence was confirmed in various nations, such as the United States, Italy, Germany, Sweden, Austria, and England (Table 1).

Second, GFAP is a highly brain-specific protein. The blood of healthy individuals has a very low level of GFAP protein [71,72]. In the brain of patients with AD, gliosis is marked by an increase in activated microglia and reactive astrocytes near the sites of Aβ plaques [73,74], and astrocyte disruption results in the easy release of GFAP from the tissue into the blood [75]. This is also supported by a plethora of evidence highlighting that the integrity of the blood–brain barrier is abnormal in AD, resulting in the microvascular leakage of proteins into the blood [76]. To determine the accuracy of blood GFAP in discriminating between individuals who are PET positive and those who are PET negative using specific Aβ, the area under the curve (AUC) was calculated and ranged from 0.69 to 0.86, indicating GFAP’s greater discriminatory power [32,42,53]. Therefore, it is possible to detect elevated blood GFAP levels as a useful diagnostic indicator of AD [77,78].

Third, a detection system including GFAP may become a tracking tool for the disease progression course and the prognosis and diagnosis of AD. In other words, this biomarker could track the therapeutic effect of developed AD therapeutic agents or the severity of AD in patients. Because inflammation occurs in pathologically vulnerable AD regions, it is possible to develop anti-inflammatory therapeutics for AD or delay the progression and onset of this ruinous disorder [79]. Therefore, blood GFAP may be a good candidate for AD diagnosis and prognosis.

Fourth, blood GFAP levels can be useful in detecting MCI and AD. In a clinical study, the increase in serum GFAP levels correlated with the MMSE score, extensively used to measure cognitive impariment [33,73]. Plasma GFAP can predict the subsequent conversion of MCI to AD [5,44]. Plasma GFAP concentrations have been shown to correlate strongly with decreased cognitive function [5,33,80]. Another review mentioned that blood GFAP was useful as an additional marker for the early detection and prediction of the time course of AD [81] and as a biomarker to predict MCI-to-dementia conversion [50].

This review found a meaningful trend in the blood GFAP concentrations between the controls and patients with MCI and AD though the result was not statistically significant (*p* = 0.050) (Figure 3). It may be demonstrated that blood GFAP should be considered for a more robust determination of the Aβ burden in cognitively unimpaired people [36].

Nevertheless, the challenges of using GFAP as an AD blood biomarker should be considered. GFAP is a commonly used marker of astroglial activation because of its markedly increased expression in most pathological conditions, including neurodegeneration and injuries [82,83]. Elevated blood GFAP concentrations have been observed not only in AD but also in various other neurodegenerative and non-neurodegenerative neurological conditions [84,85,86]. Blood GFAP levels are correlated with the clinical severity and extent of intracranial pathology in spinal cord disorders, acute CNS trauma, ischemia, neurodegenerative diseases, malignant brain tumors, and cerebrovascular events [49,82,87,88]. Therefore, it is important to determine the specificity of GFAP-related AD pathology.

In addition, we cannot assert that GFAP is more attractive than other well-known candidates such as Aβ, tau, APP, NFL, or BDNF, because research comparing their effectiveness as biomarkers is scarce. Some researchers have enumerated the effects of various proteins rather than suggesting their superiority [55,89]. A solution to this dilemma is as follows: the present trend is a mix of some candidates for AD diagnosis with or without GFAP. A few mixtures might be useful to discriminate AD: a combination of plasma p-tau181, NFL, and GFAP [62], and a combination of Aβ misfolding and GFAP [39]. Furthermore, co-detecting Aβ42/Aβ40, p-tau181, and ApoE4 improved the areas under the curve significantly (0.90 to 0.93; *p* < 0.01) in Aβ-positive versus Aβ-negative participants [51].

This study had some limitations. First, we obtained limited results because we only used data from the papers in this study. Second, our results included the control and AD groups regardless of AD stage. Therefore, further research is required to analyze the stages of AD and MCI. Third, this study included overlapping authors and cohort data among the selected articles; therefore, it needs to be considered in the interpretation of the results. Nevertheless, our study demonstrated that the levels of GFAP were remarkably changed in the blood of patients with AD. Therefore, GFAP could be a potential biomarker for diagnosis, prognosis prediction, and progression evaluation of AD, especially in relation to brain damage and cognitive impairment. As research advances, using GFAP as a blood biomarker for AD may become a valuable tool for diagnosing this devastating disease.

## Figures and Tables

**Table 1:** Characteristics of selected studies.

Author, Year	Country (Cohort)	Analyzed Group	N (M/F or Female, %)		Age (Mean (SD or Range or IQR))		Blood	GFAP (Mean (SD or IQR))	
			Control	Case	Control	Case		Control	Case
Asken, 2021 [35]	USA	CN/MCI (Cohort1)	39 (78%)	11 (22%)	72.7 (6.3)	70.7 (8.6)	Plasma	183 (140, 242),	213 (168, 254)
		CN/MCI/AD (Cohort2)	32 (45%)	MCI: 18 (25%)/ AD: 21 (30%)	75.4 (4.6)	MCI: 70.4 (11.2)/AD: 68.9 (11.2)	Plasma	110 (75, 184)	MCI: 172 (151, 233)/AD: 167 (137, 265)
Benedet, 2021 [32]	Canada (TRIAD)	CU Aβ−/CU+ Aβ+/MCI Aβ+/AD	CU Aβ−: 114 (41/73)/CU Aβ+: 42 (13/29)	MCI Aβ+: 39 (18/21)/AD: 45 (24/21)	CU Aβ−: 69.9 (9.4)/CU Aβ+: 4.1 (7.7)	MCI Aβ+: 71.2 (7.7)/AD: 66.1 (9.7)	Plasma	CU Aβ−: 185.1 (93.5)/CU Aβ+: 285.0 (142.6)	MCI Aβ+: 332.5 (153.6)/AD: 388.1 (152.8)
		Aβ− Aβ+						Aβ−: 185.1 (95.5)	Aβ+: 285.0 (142.6)
	Spain (ALFA)	CU−/CU+	CU−: 249 (96/153)/CU+: 135 (54/81)		CU−: 60.5 (4.50)/ CU+: 62.2 (4.9)		Plasma	CU−: 121.9 (42.4)/CU+: 169.9 (78.5)	
	France (BioCogBank)	CU Aβ−/MCI Aβ+/AD	21 (7/14)	MCI Aβ+: 42 (16/26)/AD: 76 (39/47)	64.4 (9.5)	MCI Aβ+: 72.4 (7.9)/AD: 72.2 (8.4)	Plasma	161.2 (67.1)	MCI Aβ+: 368.6 (158.5)/AD: 376.4 (179.6)
Benedet, 2022 [36]	USA/Canada (ADNI)	Aβ−/Aβ+	58 (34/24)	60 (34/26)	70.8 (66.5, 75.7)	73.8 (69.9, 77.4)	Plasma	113 (80.7, 154)	164 (125, 223)
Benussi, 2020 [37]	Italy	HC/AD	61 (20.6%)	63 (31.7%)	65.5 (12.3)	75.5 (8.1)	Serum	183.1 (93.7)	394.8 (176.2)
Bettcher, 2022 [38]	USA	Asymptomatic/Symptomatic (MCI/AD)	69 (21/48)	45 (23/22)	69.5 (6.4)	71.7 (7.5)	Plasma	148.1 (72.7)	265.9 (125.6)
Beyer, 2022 [39]	Germany (ESTHER)	Con/AD (within 17 y)	240 (114/126)	68 (25/43)	66.1 (4.6)	68.8 (4.3)	Plasma	99.6 (46.7)	159.0 (111.1)
Chatterjee, 2021a [40]	Australia (KARVIAH)	Aβ−/Aβ+	63 (18/45)	33 (13/20)	77.41 (5.45)	79.64 (5.20)	Plasma	151.42 (58.49)	240.12 (124.88)
		Non-SMC Aβ−/Non-SMC Aβ+/SMC Aβ-/SMC Aβ+	Non-SMC Aβ−: 14/Non-SMC Aβ+: 8	SMC Aβ−: 49/SMC Aβ+: 25				Non-SMC Aβ−: 146.83 (61.58)/Non-SMC Aβ+: 202.28 (63.81)	SMC Aβ−: 152.73 (58.18)/SMC Aβ+: 252.22 (137.75)
Chatterjee, 2021b [41]	Australia (KARVIAH)	Aβ−/Aβ+	67 (19/48)	33 (13/20)	77.78 (5.56)	79.00 (5.44)		146.96 (49.48)	211.39 (86.04)
Chatterjee, 2022 [42]	Australia (AIBL)	SMCs Aβ−/SMCs Aβ+	SMCs Aβ−: 52 (77.61%)	SMCs Aβ+: 24 (72.72%)				SMCs Aβ−: 147.85 (47.07)	SMCs Aβ+: 229.50 (93.49)
Chouliaras, 2022 [43]	UK (NIMROD/AMPLE/MIDAS/MILOS)	Con/MCI + AD	73 (43/30)	63 (43/20)	70.2 (7.79)	73.9 (7.80)	Plasma	154 (96.5)	243 (99.9)
		LBD Aβ−/LBD Aβ+	30 (25/5)	29 (23/6)	73.9 (6.25)	75.2 (6.75)		179 (62.0)	219 (85.9)
Cicognola, 2021 [44]	Sweden	Stable MCI Aβ−/Stable MCI Aβ+/MCI-AD Aβ+/MCI-other Aβ−/MCI-other Aβ+	Stable MCI Aβ−: 58 (55%)/MCI-other Aβ-: 25 (44%)	Stable MCI Aβ+: 21 (48%)/MCI-AD Aβ+: 47 (75%)/MCI-other Aβ+: 9 (33%)	Stable MCI Aβ−: 69 (8)/MCI-other Aβ−: 73 (7)	Stable MCI Aβ+: 69 (6)/MCI-AD Aβ+: 76 (7)/MCI-other Aβ+: 74 (6)	Plasma	Stable MCI Aβ−: 36 (17)/MCI-other Aβ−: 42 (16)	Stable MCI Aβ+: 46 (25)/MCI-AD Aβ+: 67 (24)/MCI-other Aβ+: 52 (11)
Ebenau, 2022 [45]	Netherlands (ADC)	Stable/Progression	337 (196/141),	64 (38/26)			Serum	190.8 (124.9)	281.1 (128.6)
Frontera, 2021 [46]	USA (ADRC)	Normal/MCI/AD (COVID-19 patients)	54 (19/35)	MCI: 54 (11/43)/AD: 53 (21/32)	71 (65–76)	MCI: 77 (70–86)/AD: 82 (72–88)	Plasma	111.4	MCI: 152.5/AD: 257.1
Gonzales, 2021 [47]	USA (TARCC)	CU/MCI/Dementia (Hispanic)	711 (196/515)	MCI: 325(102/223)/Dementia: 157 (55/102)	63 (8)	MCI: 70 (9)/Dementia: 75 (8)	Serum	134 (98, 186)	MCI: 174 (120, 249)/Dementia: 279 (182, 432)
		CU/MCI/Dementia (Non-Hispanic)	184 (67/117)	MCI: 115 (62/52)/Dementia: 351 (162/189)	72 (8)	MCI: 73 (9)/Dementia: 75 (9)	Serum	206 (145, 367)	MCI: 253 (175, 380)/Dementia: 429 (308, 591)
Gonzales, 2022 [48]	USA (TARCC)	CU/MCI/Dementia	479 (125/354)	MCI: 207 (67/140)/Dementia: 59 (20/39)	63 (7)	MCI: 71 (8)/Dementia: 74 (8)	Serum	136 (102, 189)	MCI: 179 (123, 261)/Dementia: 223 (160, 391)
Mila-Aloma, 2022 [49]	Spain (ALFA+)	Aβ−/Aβ+	262 (61.8)	135 (60)	60.6 (4.45)	62.2 (4.91)	Plasma	122 (42.8)	170 (78.5)
Oeckl, 2022 [50]	Portugal/Netherlands/Germany	Con/MCI-AD/AD	129 (66/63)	MCI-AD: 111 (47/64)/AD: 230 (91/139)	Con: 63 (57–69)	MCI-AD: 71 (64–74)/AD: 69 (62–76)	Serum	167 (108–234)	MCI-AD: 300 (232–433)/AD: 375 (276–505)
Oeckl, 2019 [33]	Germany	Con/AD	34 (25/9)	28 (9/19)	66 (57–74)	71 (67–78)	Serum	157 (126–218)	376 (294–537)
Palmqvist, 2022 [51]	Germany (Panel A+ study)	Aβ−/Aβ+	117 (65/52)	110 (48/62)	63.6 (10.8)	69.5 (7.9)	Plasma	98.9 (86.3)	155 (94)
	Sweden (Swedish BioFINDER)	Aβ−/Aβ+	403 (187/216)	290 (137/153)	71.9 (5.6)	73.0 (5.3)		89.6 (59)	130 (67)
Parvizi, 2022 [52]	Austria	HC/MCI	44 (20/24)	63 (34/29)	61.2 (55.8, 69.5)	69.9 (59.3, 77.8)	Plasma	79 (53.7, 120.6)	167.5 (93.8, 256.3)
		HC/AD		60 (24/36)		69 (61.3, 75)			181.9 (129.6, 269.6)
Pereira, 2021 [53]	Sweden (Swedish BioFINDER-2)	CU Aβ−/CU Aβ+/CI Aβ+/CI Aβ−	CU Aβ−: 217 (98/119)/	CI Aβ−: 63 (36/27)	63.8 (41.2–87.9)	67.9 (45.2–83.4)	Plasma	179.6 (31.1–534.9)	166.9 (24.5–476.0)
			CU Aβ+: 71 (35/36)	CI Aβ+: 78 (34/44)	72.1 (51.0–88.7)	73.0 (53.7–93.3)		252.1 (86.1–672.9)	262.6 (94.0–650.7)
Pichet Binette, 2022 [54]	Sweden	Non-progression to AD/Progression to AD	84 (46/38)	26 (12/14)	71.52 (8.20)	74.77 (8.12)	Plasma	164.69 (117.31)	251.30 (115.21)
Prins, 2022 [55]	Netherlands	Aβ−/Aβ+	50 (29/21)	50 (33/17)	71.88 (4.45)	73.4 (4.72)	Plasma	134.0 (50.71)	195.1 (87.13)
Salvadó, 2022 [56]	Spain (ALFA+)	Aβ−Tau−/Aβ+Tau−/Aβ+Tau+	Aβ−Tau−: 202 (72/130)	Aβ+Tau−: 88 (39/49)/Aβ+Tau−: 24 (7/17)	Aβ−Tau−: 60.5 (4.3)	Aβ+Tau−: 61.7 (5.1)/Aβ+Tau−: 64.3 (4.7)	Plasma	Aβ−Tau−:121 (42)	Aβ+Tau−: 153 (66)/Aβ+Tau+: 201 (53)
Shir, 2022 [57]	USA	Aβ−/Aβ+	99 (57/42)	101 (44/57)	75 (9)	81 (8)	Plasma	134 (65)	197 (83)
Simrén, 2021 [58]	Finland/Italy/Greece/UK/Poland/France (Add NeuroMed)	CU/MCI/AD	CU: 99 (46/53)	MCI: 107 (51/56)/AD: 103 (40/63)	CU:73 (6.14)	MCI: 74.47 (5.89)/AD: 76.35 (5.76)	Plasma	CU:125.23 (73.76)	MCI: 147.81 (81.14)/AD: 219.04 (136.1)
Simrén, 2022 [59]	Sweden	Aβ−/Aβ+	28 (17/11)	21 (9/12)	73.5 (62.8–76.3)	73 (67.0–76.0)	Serum	160 (115–282)	231 (167–283)
Stevenson-Hoare, 2022 [60]	UK (ADCC)	Con/AD	508 (221/287)	1439 (748/691)	82.2 (6.72)	68.1 (8.03)	Plasma	196 (85.3)	215 (103)
Stocker, 2022 [61]	Germany (ESTHER)	Con/AD (0–17 y)	507 (229/278)	145 (56/89)	61.2 (6.5)	66.7 (5.2)	Plasma	87.0 (46.7)	133.3 (86.0)
Thijssen, 2022 [62]	Netherlands (Amsterdam Dementia)	Con/AD (Cohort1)	40 (20/20)	40 (20/20)	56 (53–59)	58 (55–59)	Plasma	534 (342–693)	1580 (1091–1970)
		Con/AD (Cohort2)	38 (18/20)	38 (18/20)	63 (59–66)	63 (59–67)		66.6 (47.1–85.9)	119 (99.4–178)
Verberk, 2020 [63]	Netherlands (Amsterdam Dementia)	Aβ−/Aβ+	76 (49/27)	176 (89/87)	61 (9)	63 (7)	Plasma	96 (53)	168 (77)

Healthy controls (HC), subjective memory complainers (SMC), compressed subjective memory complainers (SMCs), semantic variant primary progressive aphasia (svPPA), clinically normal (CN), mild cognitive impairment (MCI), Alzheimer’s disease (AD), cognitively unimpaired (CU), glial fibrillary acidic protein (GFAP), Aβ-negative cognitively unimpaired (CU−), Aβ-positive cognitively unimpaired (CU+), Aβ-positive mild cognitive impairment (MCI+), control (Con), age: years, GFAP: pg/mL.

## Data Availability

The data supporting the findings of this study are available from the corresponding authors upon reasonable request.
